# Formerly considered rare, the ant species *Cryptoponeochracea* (Mayr, 1855) can be commonly detected using citizen-science tools

**DOI:** 10.3897/BDJ.10.e83117

**Published:** 2022-05-27

**Authors:** Ferenc Báthori, Tamás Jégh, Sándor Csősz

**Affiliations:** 1 Evolutionary Ecology Research Group, Institute of Ecology and Botany, Centre for Ecological Research, Vácrátót, Hungary Evolutionary Ecology Research Group, Institute of Ecology and Botany, Centre for Ecological Research Vácrátót Hungary; 2 Independent Researcher, Budapest, Hungary Independent Researcher Budapest Hungary; 3 MTA-ELTE-MTM Ecology Research Group, Eötvös Loránd University, Budapest, Hungary MTA-ELTE-MTM Ecology Research Group, Eötvös Loránd University Budapest Hungary

**Keywords:** ants, Ponerinae, species distribution, species monitoring

## Abstract

Citizen science is a valuable tool for monitoring different species, especially in cases concerning truly rare and difficult-to-detect species where time-consuming field studies are limited and long-term research projects are uncertain. To better understand the distribution of the rarely collected *Cryptoponeochracea* (Mayr, 1855) (Hymenoptera, Formicidae) in Hungary, we obtained the occurrence data with photos uploaded by non-professionals to the page of the largest Hungarian Facebook group dealing with ants and a citizen-science website dealing with biological data collection. In this article, we expand the known distribution of *C.ochracea* to include 46 new records from Hungary and one from Serbia. With two historical records, this previously undersampled species has now been found 48 times in Hungary. Our results prove that social media platforms and other websites for citizen science projects offer new and useful opportunities for researchers to involve non-professionals in scientific work and, thus, obtain large amounts of valuable data, even for understudied arthropod species.

## Introduction

Our knowledge on general patterns, for example, the distribution and absolute and relative abundance of a species, depends largely on collection efforts in a given area targeting the particular taxon. Species distribution and abundance provide biogeography and community ecology with indispensable information which furthers our understanding of how communities are organised and which may also help explain more general patterns underlying the structures of communities. Beyond dominant and abundant species [according to the D.A.F.O.R. scale (Morris and Therivel 2001)] that are easy to find, identify and register, many rare or occasional species are likely not to be reported on due to their hidden lifestyle, actual rarity or their aberrant phenology, which is unusual in the given taxonomic group. Rarity thus can be a self-amplifying phenomenon which may lead to the complete omission of a species from the communities’ species-pool.

In recent decades, the spread of digitisation has made several new tools available to scientists and, with the spread of internet access and smart devices, scientists can easily involve non-professionals in scientific projects (Hand 2010). Citizen science can contribute to efforts to document species in general, but it also may be applied particularly to efforts to provide data concerning invasive, rare or poorly documented species (Bodilis et al. 2013, Maistrello et al. 2016, Moulin 2020, Tiralongo et al. 2020, Marcenò et al. 2021, Mori et al. 2021). As many previous examples confirm, the many non-professionals involved can provide a wealth of interesting data for researchers (Dickinson et al. 2012, Kosmala et al. 2016, McKinley et al. 2017). Due to scientific communication (SciComm) and the growing popularity of ants as pets, more and more people are interested in ants and this creates a useful opportunity for researchers to involve these non-professionals in scientific projects. Data collection through citizen-science projects and social media groups is already common practice for many taxa in many aspects (Dickinson et al. 2012, La Sorte and Somveille 2019, Marcenò et al. 2021), but is still relatively new to the study of ants. Previously, it was used only a few times in diversity (Braschler 2009, Lucky et al. 2014, Sheard et al. 2020) and species monitoring (Castracani et al. 2020, López-Collar and Cabrero-Sañudo 2021, Schifani et al. 2021, Sorvari 2021). It has been succesfully applied to monitor the influx of new ant species in Denmark (Sheard et al. 2020), to better understand the distribution of red wood ants (*Formica* spp. Linnaeus, 1758) in Finland (Sorvari 2021) and as part of citizen-science school projects (Braschler 2009). Although some citizen-science-like projects, such as BWARS (Bees, Wasps & Ants Recording Society), have been involved in the biological data collection of Hymenoptera (including ants) for a long time, the people taking part in these projects are typically experts, such as museum and university professionals.

**Figure 1.**
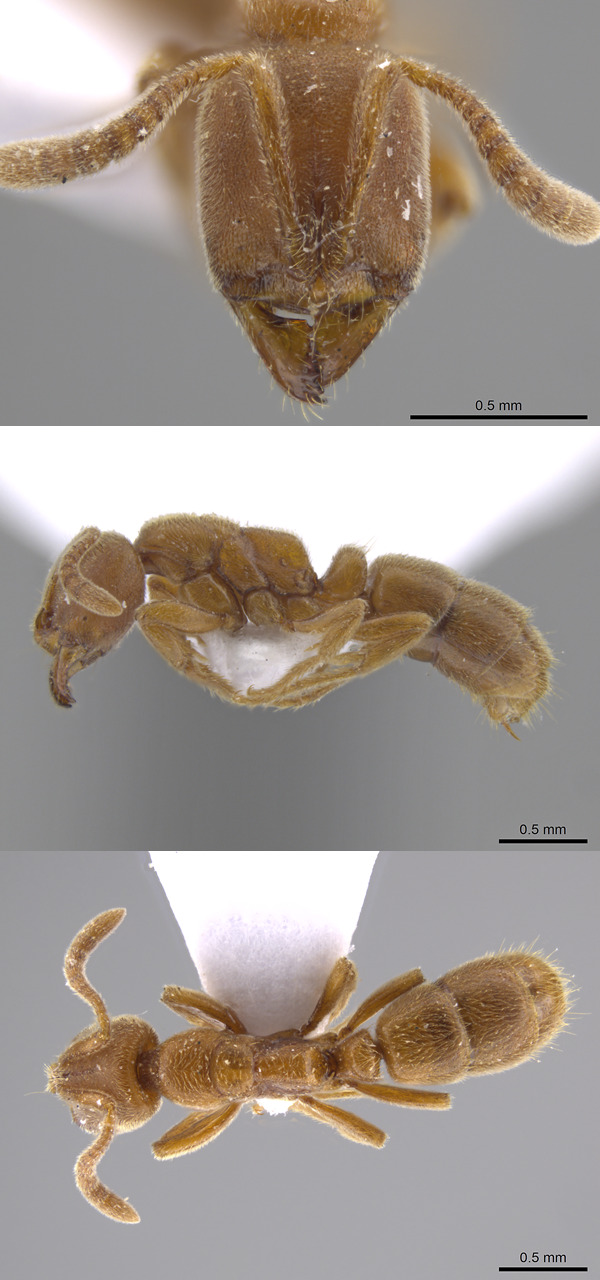
Worker of *C.ochracea* from AntWeb.org database. Specimen ID: CASENT0637778; Photographer: M. Pierce.

Although ants are dominant in the terrestrial ecosystem, occurring in large numbers in most habitats, many of their species (and particularly the rare ones) are not well documented, even in Europe, that have been under study for a long time (Espadaler and López-Soria 1991, Markó and Csősz 2001, Schifani and Alicata 2018). The tiny, endogeic (i.e. exhibiting an underground life cycle) ants are widely considered extremely rare in every European country, including Hungary, as they often cannot be collected using the most common collection methods, such as pitfall traps, baits or vacuum insect collectors (D-vac) (Romero and Jaffe 1989, Przybyszewski et al. 2020, Salata et al. 2020). In order to collect these species, typically one must dig into the soil and search for it by hand and this is time consuming and often impractical. In case of most European hypogeic ants, swarming sexuals (alate queens and males) are available for a short period of time in late summer or early autumn. *Cryptoponeochracea* (Fig. 1) swarms usually from 5-20^th^ September, *Poneracoarctata* (Latreille 1802) from 13^th^ August - 22^th^ September, *P.testacea* Emery 1895 from 3^th^ - 23^th^ September and *Proceratiummelinum* (Roger 1860) from 23^th^ August to 12^th^ September (Seifert 2018). This narrow time interval is not an ideal period for ant faunistic surveys, given that full-time myrmecologists are quite busy in this period. For myrmecologists working at an academy and higher education, this period is burdened with a significant amount of administrative and educational work. This problem can be effectively addressed by involving the general public in a citizen-science project which can further the collection of data.

The ant genus *Cryptopone* has a cosmopolitan distribution (Fernandes and Delabie 2019, Branstetter and Longino 2022) with 26 small species and the known distribution of these species is typically indicated by very sparse occurrence data concerning several geographic regions (Janicki et al. 2016, Guénard et al. 2017). To the best of our knowledge, only one species [*Cryptoponeochracea* (Mayr, 1855)] has been identified in Europe so far. We selected this species based on: (i) its extreme rarity in the Hungarian fauna and (ii) the ease with which it can be identified even through the use of images. Based on pictures, a myrmecologist familiar with the Central European ant fauna can easily identify the species. Nevertheless, only two historical records of this species are known in Hungary. Despite its wide distributional range in the Palearctic realm, the occurrence of *C.ochracea* appears to be quite scattered (Janicki et al. 2016, Guénard et al. 2017), but this is probably due to the fact that individuals are often overlooked rather than because the distribution pattern is actually as scattered as it seems, based on the little available data.

We wanted to determine whether this species is, in fact, rare or is just under-represented in faunas by using citizen-science platforms, i.e. the page of the largest Hungarian amateur myrmecologist Facebook group and one of the largest online Hungarian entomologist websites.

## Materials and Methods

### Data sources

The izeltlabuak.hu (www.izeltlabuak.hu) webpage provides a platform where amateur naturalists, nature photographers and researchers can share their data about arthropods of Hungary with one another. The website allows people to record detection data and these data can be confirmed by the contributing professionals. Observations submitted by non-professionals can be useful for research. This database is publicly available. More than 200,000 occurrence data concerning 13,701 species are currently recorded on the site, making it one of the largest Hungarian databases dealing with biological data collection and a valuable source of biology data for researchers.

The Hangya, hangyafarm Facebook group (www.facebook.com/groups/hangyaszat/) was formed on 24 July 2015 and it is now the largest Hungarian Facebook group dealing with ants (i.e. it has the most members). Currently, the group has more than 1,780 members. It mainly focuses on ant keeping, ant species and formicariums, including advice, ant determination, important information and interesting photos and videos. Data uploaded on these Hungarian groups do not necessarily show Hungarian samples only, so by examining all the uploaded data, new occurrences can also be found abroad.

### Data collection

First, we retrospectively collected data about *C.ochracea* (Mayr, 1855) from the two above-mentioned online groups. Based on the images uploaded by the members, the species identification of the individuals has been verified. The members who took the photographs were contacted and the following details were recorded: date on which a given photograph was taken, the sex of ants, location (GPS coordinates), elevation (metres above sea level) and voucher images were also requested for every individual record. After retrospective data collection, on 4 October 2020, we posted an announcement on the page of the Hangya, hangyafarm Facebook group that we were looking for species occurrence data. In this post, we shared basic information about the species (nuptial flying period, characters that could be considered for species identification) with members of the group.

We conducted a survey to identify the locations of the members of the Facebook group to determine what coverage we have for the country, thus ruling out the possibility of sampling bias. In order to do this, we listed all the members of the Facebook group and then collected their public residences (only city names that are publicly visible to everyone) in Hungary, if this information were available. We also collected coordinates and elevations based on residences (cities). Furthermore, we also assessed the number of active and passive members in the group who had a public residence. Active members were those who shared pictures, data or asked questions related to ants. Inactive members were those who had not shown activity in the group since joining. We no longer used residence data for any other activity.

Samples were identified by images whenever possible, based on their quality, otherwise the observers were asked to collect and send voucher specimens. The species of a given sample was determined by author SC. The specimens are deposited in SC’s private collection at Eötvös Loránd University (Budapest, Hungary). Female castes, queens and workers were identified. Males were excluded from the pool of samples, as it is hard to identify them on the basis of photos.

## Results

**Figure 2.**
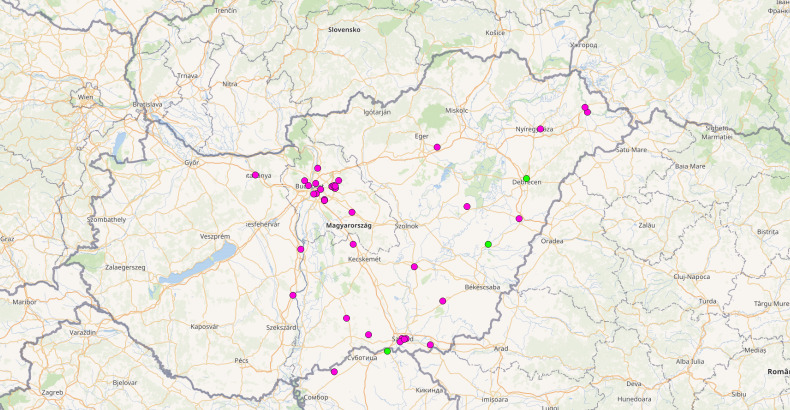
New (magenta circles) and historical (green circles) occurrence data (Petrov and Collingwood 1992, Csősz 2003) of C.ochracea in Hungary and Serbia. The map was created by using the QGIS Desktop software (ver. 3.10.6, http://www.qgis.org).

A total of 47 new occurrence records have been collected from the two online platforms since the start of the study (Fig. 2), represented by altogether 265 identified *C.ochracea* individuals, of which 48 were workers and 217 were alate queens. Forty six occurrence records have been collected from the Hangya, hangyafarm Facebook group and one from the izeltalbuak.hu website. The 47 new records were provided from different parts of Hungary (46 occurrences) and Serbia (one occurrence). The date of the first record is 3 October 2016 and the date of the last record is 28 September 2021 (Table 1). Uploaders were also urged to provide coordinates for samples and this helped us to obtain a more accurate picture of the distribution of this species in Hungary.

**Figure 3.**
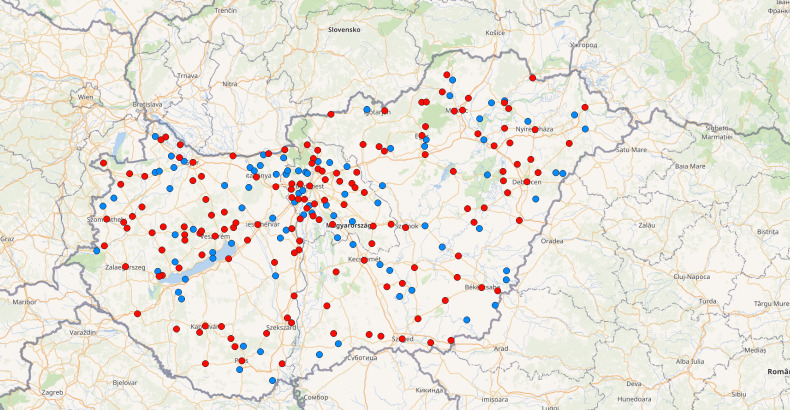
Distribution of active (red circles) and inactive (blue circles) group members based on their public residences. The map was created by using the QGIS Desktop software (ver. 3.10.6, http://www.qgis.org).

We found the residential addresses of a total of 634 of the Facebook group members publicly listed in their profiles. We found that many members were concentrated in the capital (Budapest: 172 members) and larger cities (Debrecen: 34, Pécs: 15, Miskolc: 14, Szeged: 13, Győr: 12, Nyíregyháza: 10, Sopron: 9, Kecskemét: 7, Székesfehérvár: 6) (Suppl. material 1). However, the other members of the group were scattered throughout the country, thus nicely covering almost all regions and different habitats of Hungary (Fig. 3). Based on their posts in the group, it can be seen that there are a negligible number of members living abroad, but the residences are not public for most group members. One exception is the uploader of data from Bajom, Serbia. Based on their activity in the group, more than half of the members (339 of 634) with a public residence are active, sharing pictures and information about ants. Ant individuals were found between 75 (Szeged, Hungary) and 274 (Hűvösvölgy, Hungary) metres above sea level. The average altitude of the new occurrence data is 129.91 m a.s.l. The highest settlement where a group member lives is Zirc (399 m a.s.l.), while the lowest settlement was Szeged (75 m a.s.l). The average altitude of the residences of the group members was 139.94 m a.s.l.

## Discussion

Our results confirm that the data collected by non-experts can be valuable for efforts to monitor supposedly rare species and a large amount of data can be collected in a relatively short time. With our new records, the number of available distribution data concerning *C.ochracea* has now been increased from two to 48 in Hungary (Csősz 2003) and one from two in Serbia (Petrov and Collingwood 1992) (Fig. 2). Furthermore, in the Facebook group dealing specifically with ants, data collection proved to be more efficient than on izeltlabuak.hu, which is probably because all members deal with ants here, while fewer people deal specifically with ants on izeltlabuak.hu. Previously, this species was thought to be quite rare in the region (Seifert 2018), but the results showed that it was under-represented due to the small number of data collectors. Until the present research undertaking, this species was known only from the eastern region of Hungary, mainly from lowland areas (Csősz 2003), but the current results extend the known distribution to the central areas of Hungary, all the way to the Danube River.

Interestingly, even though the Facebook group has more than 300 active members from all over the country, the species' new occurrence data were mainly obtained from the Transtisza and Danube-Tisza Interfluve (Fig. 3). Although we do not know much about the species' ecological needs, this is probably related to the fact that *C.ochracea* is to be associated with lowland habitats in eastern and central Hungary and is not a result of sampling bias. This also seems to be confirmed because our new data usually came from areas with low altitudes, mainly below 200 m a.s.l., similar to other data on the species from neighbouring countries. *Cryptoponeochracea* is known from two sites in Romania [Bucharest (72 m a.s.l.) and Băile Herculane (137 m a.s.l.)] (Montandon 1907, Csősz 2003), two sites in Serbia [Бајмок (114 m a.s.l.] and Horgoš (84 m a.s.l.)] and another site in Slovenia [Fiesa (38 m a.s.l.] (Petrov and Collingwood 1992, Bračko 2003). Interestingly, the species occurs in the Mediterranean from coastal regions to higher mountainous areas, with the highest area at 976 m a.s.l. in Spain, 711 m a.s.l. in Italy, 1240 m a.s.l. in Greece and 1055 m a.s.l. in Turkey (Janicki et al. 2016).

The results highlight the potential uses of information provided by groups of people dealing with ants on social media to further our knowledge of ant species distributions. The study of species distributions has recently also benefitted from the spread of smart devices with better quality cameras and the use of online platforms (e.g. iNaturalist, various Facebook groups dealing with wildlife etc.), where amateurs can share their observations with one another and with scientists. Of course, the method we used cannot be applied to all ant species, pictures taken by non-professional data providers are insufficient for several ant species. However, our method is useful for easily identifiable species. It may be worthwhile to extend data collection to international Facebook groups dealing with ants in the future, thus gaining knowledge about other understudied species, possibly even in other regions and not just about their distribution. One of the largest international groups dealing with ants currently has more than 9,000 members, so the information provided by these groups may have great potential use for similar research in the future. It should also be noted that, based on our results, it appears that, in addition to applications and sites well known to scientists and developed specifically for citizen-science purposes, Facebook groups with specific topics may be much more proficient than a well-developed application like iNaturalist to record data on certain groups of organisms. It would be worthwhile for the scientific community to draw people’s attention to these specific sites as they are more accessible and transparent to researchers or to publish targeted calls for data collection in popular social media (Facebook groups, Twitter etc.).

The worldwide SARS-CoV-2 lockdowns have presented huge challenges for everyone and scientists are no exception (Korbel and Stegle 2020, Myers et al. 2020). Due to travel restrictions, curfews and budget cuts, classic species monitoring methods are difficult to implement and this encourages the development and use of new methods (Birkin et al. 2021, Dwivedi 2021). The online platforms mentioned above can be used with only minimal costs, as there is no need to pay researchers to collect the specimens and the use of social media platforms (e.g. Facebook groups, Twitter etc.) and websites dealing with biological data collection (e.g. iNaturalist) is, in most cases, free of charge (Chamberlain 2018). With these easy-to-use online platforms, researchers can access a large amount of information made accessible by a large group of data providers and they can efficiently obtain a large amount of data which can be used in many fields of sciences in a short period of time without having to use costly methods.

## Supplementary Material

B32A6182-4830-5D0B-836E-2D980C564C29 10.3897/BDJ.10.e83117.suppl1 Supplementary material 1 Formerly rare ant species *Cryptoponeochracea* (Mayr, 1855) commonly detected using citizen-science tools
Data type distribution of data providers File: oo_669389.xlsx https://binary.pensoft.net/file/669389 Ferenc Báthori, Tamás Jégh, Sándor Csősz

## Figures and Tables

**Table 1.** The new occurrence data of *C.ochracea* from Hungary and Serbia. Coordinates with high positional accuracy are marked in bold.

Date	Queen	Worker	Locality	GPS coordinates	Elevation (metres above sea level)
03.10.2016	3	0	Mezőkövesd	**47.8091°N, 20.5576°E**	115
02.09.2018	1	0	Ruzsa	**46.2873°N, 19.7408°E**	113
22.06.2019	0	1	Rákoscsaba	**47.4935°N, 19.3060°E**	148
04.09.2019	0	1	Hűvösvölgy	**47.5399°N, 18.9799°E**	274
08.09.2019	1	0	Szeged	**46.2561°N, 20.1389°E**	80
11.09.2019	1	0	Rákoscsaba	**47.4936°N, 19.3048°E**	148
15.09.2019	4	0	Pesterzsébet	47.4359°N, 19.1193°E	113
27.09.2019	1	0	Pilis	**47.2857°N, 19.5438°E**	140
28.09.2019	1	0	Karcag	**47.3319°N, 20.9133°E**	86
12.08.2020	1	0	Gyopáros	**46.5639°N, 20.6242°E**	86
20.08.2020	1	0	Paks	**46.6110°N, 18.8406°E**	122
20.08.2020	1	0	Rákoscsaba	**47.4918°N, 19.3121°E**	154
01.09.2020	1	0	Rákoscsaba	**47.4936°N, 19.3059°E**	148
01.09.2020	1	0	Rákoscsaba	**47.4941°N, 19.3155°E**	147
09.09.2020	1	0	Pécel	47.4810°N, 19.3343°E	199
10.09.2020	1	0	Tatabánya	**47.5859°N, 18.3935°E**	145
24.09.2020	1	0	Budapest	47.5187°N, 19.1121°E	113
25.09.2020	5	0	Szeged	46.2287°N, 20.1164°E	75
25.09.2020	1	0	Budapest	47.5011°N, 19.0251°E	125
25.09.2020	26	0	Pécel	**47.4795°N, 19.3451°E**	182
30.09.2020	2	0	Isaszeg	**47.5414°N, 19.3855°E**	180
02.10.2020	5	0	Pécel	**47.4795°N, 19.3451°E**	182
02.10.2020	2	0	Kiskunhalas	**46.4229°N, 19.4790°E**	125
02.10.2020	5	40	Dunaújváros	**46.9863°N, 18.9341°E**	124
02.10.2020	1	0	Dunaújváros	**46.9863°N, 18.9341°E**	124
03.10.2020	2	0	Pécel	**47.4968°N, 19.3441°E**	176
03.10.2020	85	0	Pécel	**47.4795°N, 19.3451°E**	182
03.10.2020	4	0	Rákoscsaba	**47.4936°N, 19.3059°E**	148
03.10.2020	0	3	Rákoscsaba	**47.4936°N, 19.3059°E**	148
03.10.2020	3	0	Bajmok (Serbia)	**45.9812°N, 19.3328°E**	114
04.10.2020	1	0	Nyíregyháza	47.9546°N, 21.7881°E	108
06.10.2020	1	0	Pécel	**47.5015°N, 19.3295°E**	184
11.10.2020	1	0	Csepel-Szabótelep	**47.4329°N, 19.0880°E**	99
12.07.2021	0	3	Budapest	**47.4704°N, 19.1706°E**	139
05.08.2021	1	0	Gyál	47.3808°N, 19.2157°E	115
05.08.2021	1	0	Vásárosnamény	**48.0880°N, 22.3470°E**	110
30.08.2021	1	0	Berettyóújfalu	47.2339°N, 21.5339°E	92
30.08.2021	10	0	Gyál	47.3808°N, 19.2157°E	115
31.08.2021	1	0	Kunszentmárton	**46.8436°N, 20.2860°E**	83
04.09.2021	1	0	Dunakeszi	**47.6405°N, 19.1352°E**	123
04.09.2021	1	0	Szeged	**46.2552°N, 20.1872°E**	83
12.09.2021	1	0	Vásárosnamény	48.1267°N, 22.3183°E	110
17.09.2021	3	0	Makó	**46.2041°N, 20.4763°E**	81
17.09.2021	1	0	Gyál	**47.3887°N, 19.2158°E**	117
18.09.2021	8	0	Gyál	47.3808°N, 19.2157°E	115
25.09.2021	1	0	Szeged	46.2500°N, 20.1666°E	79
28.09.2021	23	0	Lajosmizse	47.0264°N, 19.5577°E	137
